# Customized exercise programs implemented by physical therapists improve exercise-related self-efficacy and promote behavioral changes in elderly individuals without regular exercise: a randomized controlled trial

**DOI:** 10.1186/s12889-019-7270-7

**Published:** 2019-07-09

**Authors:** Takashi Wada, Hiromi Matsumoto, Hiroshi Hagino

**Affiliations:** 1 Rehabilitation Division, Tottori University Hospital, 36-1 Nishi-cho, Yonago, Tottori 683-8504 Japan; 2 Department of Rehabilitation, Faculty of Health Science and Technology, Kawasaki University of Medical Welfare, 288 Matsushima, Kurashiki, Okayama 701-0193 Japan; 3 School of Health Science, Tottori University Faculty of Medicine, 86 Nishi-cho, Yonago, Tottori 683-8503 Japan

**Keywords:** Customized exercise programs, Self-efficacy for exercise, Stages of change for exercise behavior, Exercise adherence, Randomized controlled trial

## Abstract

**Background:**

Specialized, individualized exercise programs that increase self-efficacy (SE) are essential for the development and maintenance of regular exercise. The objective of this study is to examine the effect of customized exercise programs (CEPs) implemented by physical therapists in improving exercise-related SE and promoting behavioral changes among elderly individuals who do not exercise regularly compared with commonly prescribed exercises.

**Methods:**

In this randomized controlled study, the sampling frame consisted of participants in an annual town-sponsored medical check-up. The inclusion criteria were no regular exercise and age of 65–74 years at enrollment. The subjects in the intervention group (CEP group) were instructed to perform individualized exercises prescribed based on an original algorithm. Data collection was performed at baseline and 3, 6, 9, and 12 months after exercise instruction. The primary outcome was SE for exercise at the last time point. Secondary outcomes were SE for exercise, stage of change in exercise behavior, knee pain, and low back pain at each evaluation time point.

**Results:**

Fifty subjects (CEP group *n* = 26; control group *n* = 24) were randomized. In the CEP group, 25 of 26 subjects were analyzed at 3 months, 26 of 26 subjects were analyzed at 6 and 9 months, and 25 of 26 subjects were analyzed at 12 months. In the control group, 23 of 24 subjects were analyzed at 3, 6, 9, and 12 months. SE for exercise improved 24.0% (CEP group 30.8%; control group 16.7%) compared to baseline. No significant differences were observed in the primary outcome. SE for exercise was significantly lower at 9 and 12 months compared with baseline in the control group. Stage of change for exercise behavior was significantly higher at 3 months compared with baseline in the CEP group and at 6 months in the control group. Knee pain was worse at 3 months compared with baseline in the control group.

**Conclusions:**

This study suggested that exercise instruction with CEPs contributes to the maintenance of SE for exercise and is useful for changing exercise behavior in elderly individuals who do not regularly exercise.

**Trial registration:**

UMIN000027240, registered on 03/05/2017.

**Electronic supplementary material:**

The online version of this article (10.1186/s12889-019-7270-7) contains supplementary material, which is available to authorized users.

## Background

Physical activity has great health-promoting benefits in older adults. Regular exercise lowers the risk of cardiovascular disease, cerebrovascular disease, hypertension, colon cancer, breast cancer, and type 2 diabetes [1]. For the elderly, increasing the amount of exercise or daily physical activity possibly leads to maintenance of motor function [2], improvement of mental health [3, 4], and prevention of dementia [5]. However, a survey of approximately 1.9 million people in 168 countries conducted by the World Health Organization showed that 27.5% of adults had levels of physical activity that were insufficient for health maintenance in 2016; this percentage was not statistically different from 28.5% in a 2001 study [6]. This report suggested that the lack of exercise in adults is a global problem. Education aimed at increasing physical activity and promoting exercise habits in adults is thus essential for preventing lifestyle diseases and improving motor function.

To develop exercise habits, support via phone calls or home visits [7], beliefs about exercise [8], and participation in group activities [9, 10] have been reported to be important. Factors such as lack of interest in exercise and physical fatigue are known to make it difficult to develop exercise habits [11]. In recent years, the relationship between self-efficacy (SE) and regular exercise has been attracting attention as increased SE is speculated to trigger actions needed to build exercise habits [12–16]. SE refers to a belief in one’s capability to successfully execute the actions necessary to satisfy specific situational demands [17]. Specifically, professional exercise instruction that increases self-confidence in performing exercise or self-esteem and exercise instruction based on individualized programs were reported to have a positive impact on SE of participants [18, 19]. These findings suggest that specialized and individualized exercise programs that increase SE are essential for the development and maintenance of exercise habits. However, commonly prescribed exercise programs tend to be standardized and unattractive. Musculoskeletal disorders such as knee pain due to osteoarthritis, low back pain [20], and joint pain due to rheumatoid arthritis are among the most common health problems in older adults. These disorders often require multidisciplinary care for confirmation of the diagnosis and drug treatment [21, 22]. However, patients with musculoskeletal disorders often receive recommendations for exercise without regard for age, pain, or physical ability. For example, in squatting, the number of sets and repetitions is often specified without consideration of age or symptoms such as pain. Exercise programs that are not individualized would prevent SE from being developed and lead to failure to start or continue exercising. On the other hand, it has been reported that customized interventions that take into account individual differences are more important for behavioral change related to physical activity than general interventions [23, 24]. Therefore, exercise programs should take into account individual differences, which may be useful for behavioral change. Nevertheless, it is not known whether actual customized exercise programs designed with consideration of individual differences could improve SE among the elderly and promote the behavioral changes that lead to exercise habits.

The objective of the present randomized controlled study is to examine the effects of an intervention consisting of customized exercise programs (CEPs) implemented by physical therapists in improving exercise-related SE, promoting behavioral changes, and maintaining exercise adherence in elderly individuals who do not regularly exercise compared with commonly prescribed exercises.

## Methods

### Study methods

This study was an open-label prospective randomized controlled study with an allocation ratio of 1:1. A statistician not affiliated with the study prepared the allocation table with a computer before the start of the study. The case enrollment center allocated subjects to treatment arms based on the allocation table in the order that they completed the Good Ageing and Intervention Against Nursing Care and Activity Decline (GAINA) study [25]. The center managed the allocation table until the end of the study. The allocation table was not disclosed to any individuals or parties other than the person making the allocation. In this study, researchers who performed the motor function testing and statistical analysis were blinded, while the physical therapists who provided exercise instructions and the elderly study participants who received the instructions were not blinded. All authors of this study also participated in the GAINA study; therefore, they were able to access the data sets generated in the GAINA study for analysis in the current study. The authors obtained written permission from the GAINA study group before using the data sets.

### Subjects

The participants of this study consisted of subjects from the GAINA study, a population-based study of residents of the town of Hino, Tottori Prefecture, Japan that began in 2014. In April 2018, the population of Hino consisted of 3,194 residents, of whom 47% were older than 65 years of age. Study participants were recruited from a group of individuals who had registered for an annual town-sponsored medical check-up. The inclusion criteria for the current study were 1) no regular exercise (defined as exercising for 30 min or longer twice a week or more frequently for at least 1 year), 2) ability to walk independently, 3) ability to undergo an exercise assessment, and 4) enrollment in the GAINA study between the ages of 65 and 74 years. The exclusion criteria were 1) locomotive syndrome, 2) sarcopenia, 3) history of serious cerebrovascular disease or heart disease, 4) history of surgery involving the trunk or lower extremities for an orthopedic condition, and 5) need for long-term care insurance as certified by the Ministry of Health, Labour and Welfare. The subject recruitment period lasted from May 31, 2017 to June 2, 2017. The subject follow-up period lasted from May 31, 2017 to May 31, 2018. Data were collected for this study after the investigation planned for the GAINA study at the community center in the town of Hino, Tottori. This study was registered in the University Hospital Medical Information Network Clinical Trials Registry (Study ID, UMIN000027240).

### Intervention

#### CEP group

An “algorithm for automatically displaying exercise methods” (Japanese patent publication number, 2018–055626) developed based on the GAINA study database was used to prescribe individualized exercises for the subjects in the CEP group. In this algorithm, physical characteristics of individuals are estimated based on their age, sex, height, body weight, knee and low back pain (assessed using visual analogue scale [VAS]), grip strength, walking velocity, bone mass, and one-leg standing time with the eyes open. Interpretation of the characteristics (comparison of measured values with age-matched mean values), musculoskeletal age (score indicative of physical condition), exercise methods suitable for the subject’s physical condition, and number of repetitions and sets for the exercises were determined. Exercise prescriptions consisted of 1 to 3 exercise methods from a list of approximately 100 exercise methods that can be implemented at home selected by the algorithm, which was implemented in a software program accessible via a personal computer. The number of repetitions and sets and the duration were adjusted.

The protocol for exercise instruction in the CEP group was as follows. First, blinded members of the research team assessed each subject’s age, sex, height, body weight, levels of knee and low back pain based on VAS, grip strength, walking velocity, bone mass, and one-leg standing time with the eyes open. Other research team members then entered the data from individual subjects into a software program on a personal computer designed based on the above algorithm. They obtained a printout with illustrations of 2 or 3 exercise programs suitable for each subject’s physical condition. The printout with illustrations of exercise programs included details about musculoskeletal age, exercise methods, and number of repetitions and sets. Members of the research team who are physical therapists gave instructions for the exercises to subjects using the printout and asked them to carry out those exercise programs every day at home. The subjects were also asked to document the dates on which they carried out the programs in an exercise calendar distributed to them.

#### Control group

The subjects in the control group were instructed by physical therapists to perform squats (5 times) and one-leg standing (1 min on each leg) 3 times a day. According to the exercise instruction protocol for the control group, members of the research team who are physical therapists gave the subjects instructions using a printout with illustrations of squatting and one-leg standing and asked them to perform these exercises every day at home. The subjects were also asked to document the dates on which they carried out the exercises in an exercise calendar distributed to them.

### Data collection technique and tools

Baseline characteristics of the subjects such as age, height, body weight, body mass index, employment status, comorbidity status (hypertension, dyslipidemia, and diabetes), and past diagnosis of knee osteoarthritis, hip osteoarthritis, osteoporosis, and lumbar spinal stenosis were collected. Grip strength was measured using a dynamometer (T. K. K. 5401, Takei Scientific Instruments, Niigata, Japan). Subjects underwent 2 measurements for each hand. The highest value was used as the representative value. Walking velocity was measured using a gait analysis system (OptoGait, Microgate, Bolzano, Italy). Subjects walked at a comfortable pace along a 7-m straight line, which included 2 m each of takeoff and slowdown portions. The speed in the middle 3-m portion was calculated using a dedicated software program. Calcaneal bone mass was measured using quantitative ultrasound (QUS) (CM-200 sonometer, Furuno Electric, Nishinomiya, Japan) with the subject in a sitting position and the right foot placed on the QUS measurement stand. Coupling gel was applied on both sides of the right calcaneus. Young adult mean (YAM) values were calculated based on the speed of sound determined by QUS. Demographic data, motor function data, and structural measurements were collected using various questionnaires or measurements from the GAINA study by blinded researchers before exercise instructions were given.

The primary outcome was SE for exercise [26]. This index consisted of 5 questions about self-confidence in exercising under each of the following conditions: physical fatigue, mental stress, lack of time, extraordinary circumstances, and bad weather (extraordinary circumstances were considered to be unrelated to SE). In response to the question, “Do you have the confidence to exercise regularly under the following conditions?,” subjects were asked to select 1 of 5 answers ranging from “No, I don’t have any confidence at all (1 point)” to “Yes, I am quite confident (5 points).” The score can range from 4 to 20. The test-retest reliability and internal validity of this index have been verified [26]. Secondary outcomes were stages of change in exercise behavior, exercise adherence, and levels of knee and low back pain. Stages of change in exercise behavior [26] consists of 5 statements describing actual past and present exercise behaviors and level of motivation (subjects’ readiness to take action) with regards to exercise behaviors. These statements are 1) “I am not exercising now and will not exercise in the future” (indifference phase), 2) “I am not exercising now, but thinking about starting within 6 months” (interest phase), 3) “I am exercising now, but not regularly” (preparation phase), 4) “I am exercising now, and it has been no longer than 6 months since I started” (execution phase), and 5) “I have been exercising regularly for at least 6 months” (maintenance phase). Subjects selected the statement that most accurately described their opinion or behavior at the time of the study. The reliability and validity of this index have been verified [26]. Subjects were asked to document the dates on which they exercised on the exercise calendar for 12 months and submit the calendar every 3 months. Exercise adherence was evaluated using the exercise calendar. Exercise adherence was defined as the frequency of exercise per 3 months, which was calculated by dividing the number of days on which a subject exercised by the number of days in the investigation period. Knee and low back pain were evaluated using VAS. Subjects were asked to mark the point on a 100-mm line that corresponds to the severity of their current pain, assuming that the left end of the line indicates no pain and the right end indicates the most severe pain. Primary and secondary outcomes except for exercise adherence were measured using self-administered questionnaires as part of baseline assessment before exercise instructions were given. In addition, researchers sent these indexes to subjects by mail at 3, 6, 9, and 12 months after exercise instructions, which were completed and returned by subjects.

### Sample size

The target number of subjects was 60, which was based on the following assumptions: difference of 4 in the primary outcome (difference in the mean score for exercise-related SE between the groups at 12 months), standard deviation of 3 in each group, and 20% missing data. This resulted in a sample size of 13 per group needed to achieve a statistical power of 80%. When the effect of the intervention was estimated to be somewhat smaller, assuming the difference in the mean values between the groups to be 2.5, the necessary sample size per group with the other assumptions unchanged was 30. Although the sample size was calculated based on the feasibility of the study, we selected a sample size of 30 per group as this was thought to provide sufficient statistical power.

### Statistical methods

The primary outcome was SE for exercise at 12 months (the last time point). Using linear mixed-effects models with the scores of SE for exercise at all 4 time points during the follow-up period (3, 6, 9, and 12 months) as the outcomes, analysis was performed with age, sex, employment status, knee pain, low back pain, and baseline score of SE for exercise as moderator variables. In order to appropriately handle the intermittent presence of missing data or dropouts during the follow-up period, modeling was performed with mixed-effect models for repeated measurements. Assuming that the correlation between outcomes at different time points is unstructured, between-group differences at each time point were evaluated using a regression model with an interaction term between group and time point. The primary analysis was performed by estimation and test of between-group differences in the score of SE for exercise at 12 months (the last time point).

Secondary outcomes consisted of SE for exercise, stage of change for exercise behavior, knee pain, low back pain, and exercise adherence at 3, 6, 9, and 12 months. Between-group comparisons at each evaluation time point were performed using the Mann-Whitney U test. Within-group comparisons of baseline values and values at each evaluation time point were performed with the Wilcoxon signed-rank test. The significance level of 5% was used.

### Ethical considerations

All of the subjects provided written informed consent. The study was approved by the ethics committee of the Faculty of Medicine, Tottori University (No. 1701B076).

## Results

### Study flowchart

**Fig. 1:**
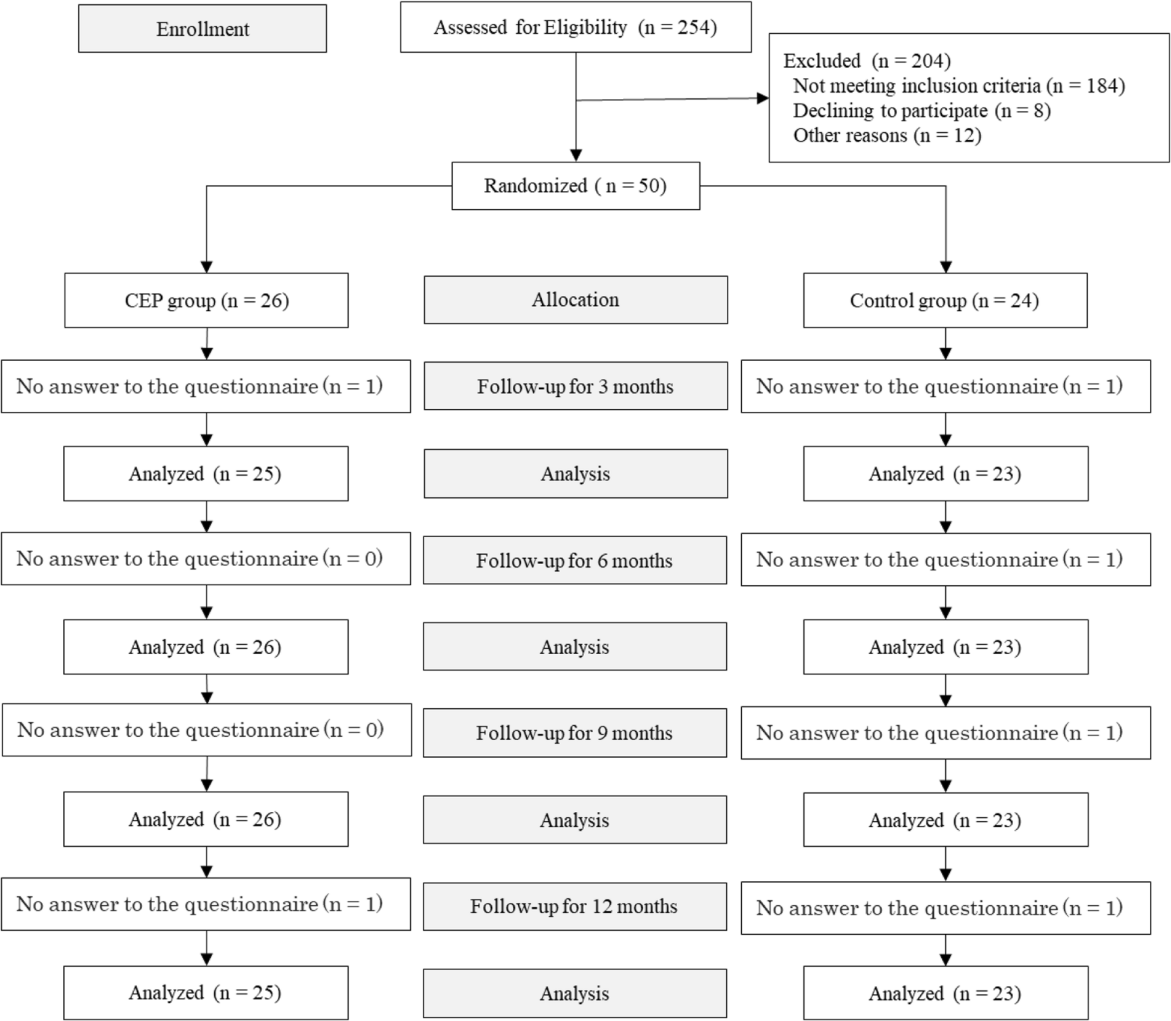
Flow diagram of the two groups’ progress through the phases of the randomized trial

The study flowchart is presented in Fig. 1. The target number of subjects was 60, but only 50 subjects actually participated in the study.

### Details of exercise in intervention group

Additional file 1 shows the exercise programs prescribed to each of 26 subjects in the CEP group.

### Baseline data

Table 1 shows the baseline data for each group. At baseline, the incidence of hypertension was significantly higher in the CEP group (*p* = 0.019). No other variables showed significant between-group differences at baseline.

**Table 1:** Baseline data

	CEP (*n* = 26)	Control (*n* = 24)	*p*-value
Age (years)	69.1 ± 2.9	68.9 ± 2.2	0.781
Gender (male/female)	11 / 15	9 / 15	0.729
Height (cm)	158.4 ± 8.2	158.8 ± 8.9	0.876
Weight (kg)	58.7 ± 11.9	55.6 ± 8.6	0.309
BMI (kg/m^2^)	23.2 ± 3.2	22.0 ± 2.2	0.120
Employed (%)	42.3	41.7	0.963
Comorbidities			
Hypertension (%)	42.3	12.5	0.019
Dyslipidemia (%)	34.6	25.0	0.459
Diabetes (%)	11.5	12.5	0.917
Musculoskeletal diseases			
Osteoarthritis of the knee (%)	11.5	8.3	0.706
Osteoarthritis of the hip (%)	0.0	0.0	1.000
Osteoporosis (%)	15.4	12.5	0.769
Lumbar spinal stenosis (%)	15.4	12.5	0.769
VAS for knee pain (mm)	0.0 (0.0–10.0)	0.0 (0.0–13.8)	0.867
VAS for back pain (mm)	0.0 (0.0–45.0)	0.0 (0.0–41.5)	0.812
Grip strength (kg)	30.4 ± 9.0	30.2 ± 8.9	0.947
Walking velocity (m/sec)	1.3 ± 0.2	1.4 ± 0.2	0.119
YAM (%)	76.7 ± 15.9	76.1 ± 11.6	0.895
Self-efficacy for exercise	11.0 (9.0–13.3)	13.0 (12.0–15.8)	0.064
Stage of change for exercise behavior	2.0 (2.0–2.0)	2.0 (2.0–2.8)	0.578

Data are presented as means ± SD or medians (interquartile range)

*BMI* Body mass index, *CEP* Customized exercise program, *VAS* Visual analogue scale, *YAM* Young adult mean

### Number of subjects analyzed

In the CEP group, 25 of 26 subjects were analyzed at 3 months, all 26 subjects were analyzed at 6 and 9 months, and 25 of 26 subjects were analyzed at 12 months. In the control group, 23 of 24 subjects were analyzed at 3, 6, 9, and 12 months because some subjects did not answer the questionnaire.

### Outcome and estimates

There were no significant differences in the variables examined between the CEP and control groups throughout the 12 months. Within-group comparisons indicated the appearance of behavioral changes at 3 months in the CEP group and at 6 months in the control group. In the CEP group, SE for exercise did not decrease and there was no exacerbation of knee and low back pain. In the control group, SE for exercise was significantly lower at 9 and 12 months and knee pain was worse at 3 months compared with baseline. Exercise adherence worsened in the CEP group after 3 months and in the control group after 9 months.

### Primary outcome

Table 2 shows the results for the primary outcome. No significant differences were observed at any time point.

**Table 2:** Mixed-effect models for repeated measurements result of self-efficacy for exercise

Time period	Estimate	Standard error	*p* value	95% CI
3 months	−0.58	1.15	0.62	−2.89–1.73
6 months	0.07	1.22	0.95	−2.39–2.53
9 months	0.15	1.11	0.90	−2.08–2.37
12 months	0.42	0.95	0.66	−1.51–2.34

*CI* Confidence interval

### Secondary outcomes

**Fig. 2:**
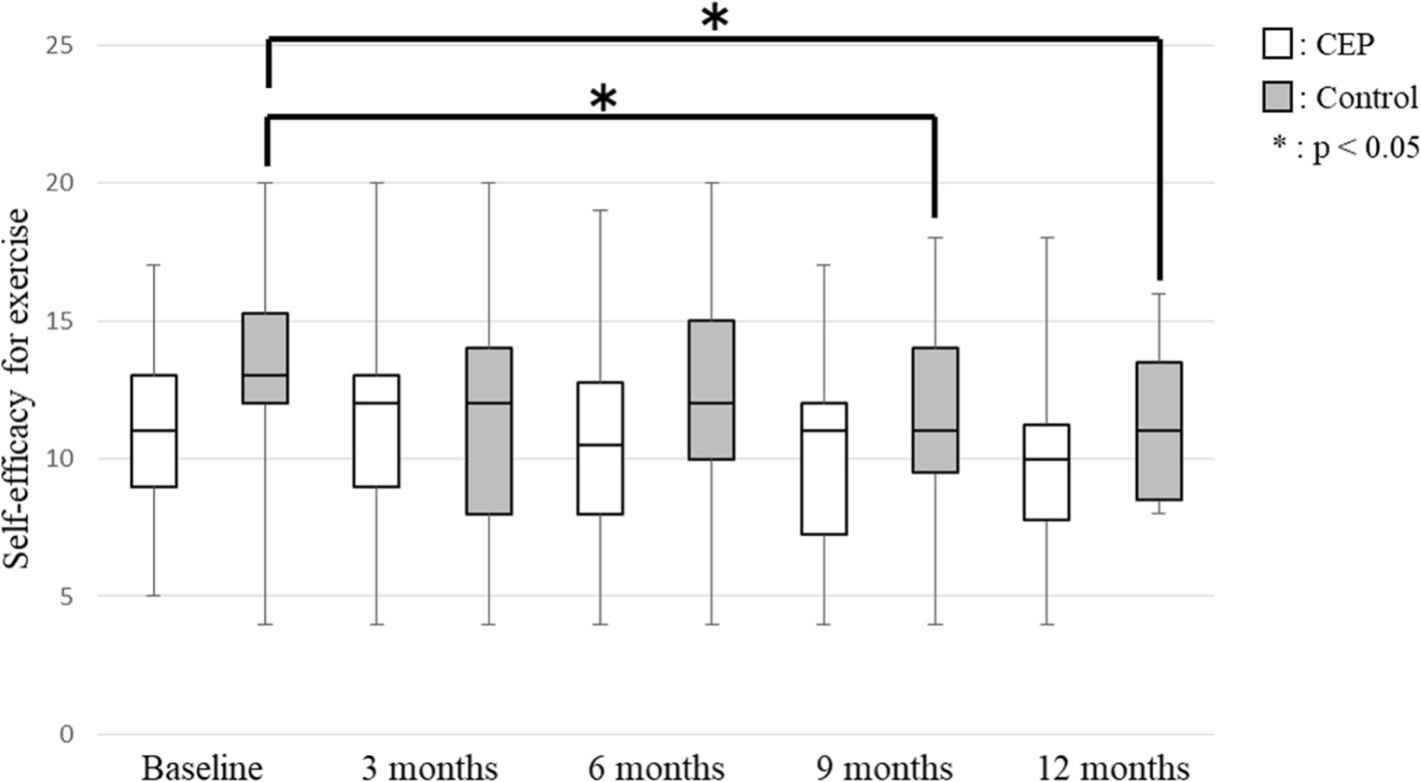
Self-efficacy for exercise

**Fig. 3:**
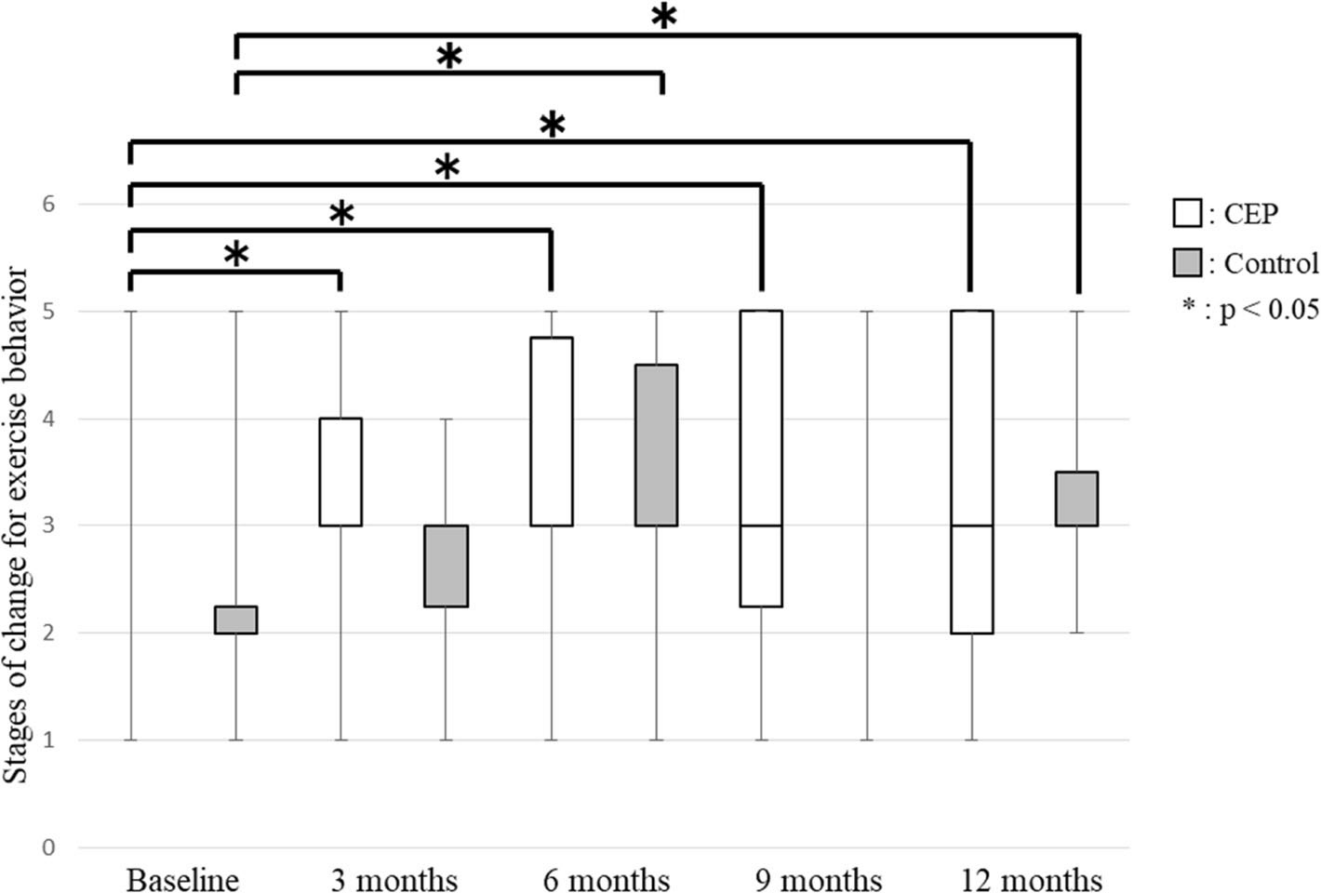
Stages of change for exercise behavior

**Fig. 4:**
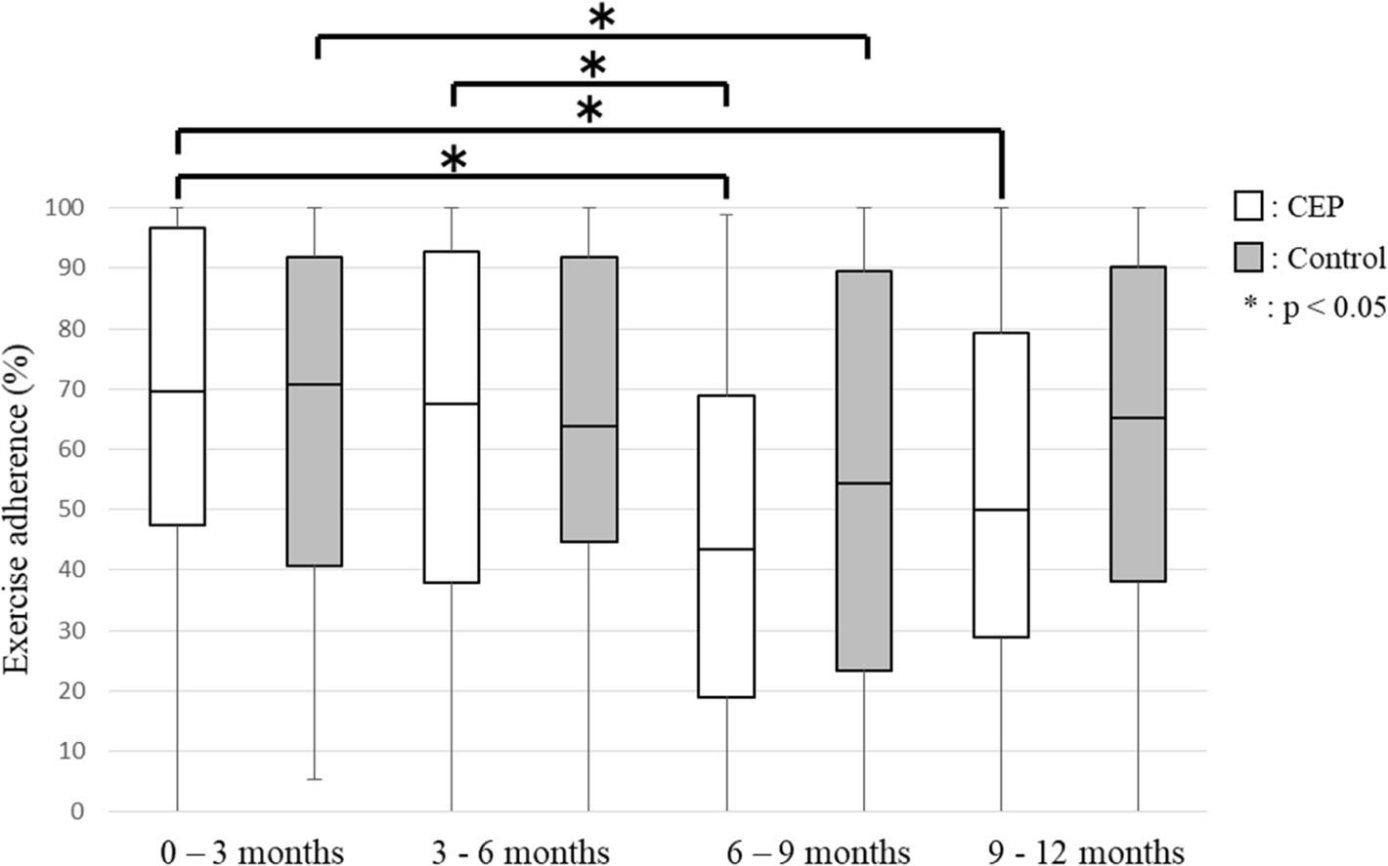
Exercise adherence

**Fig. 5:**
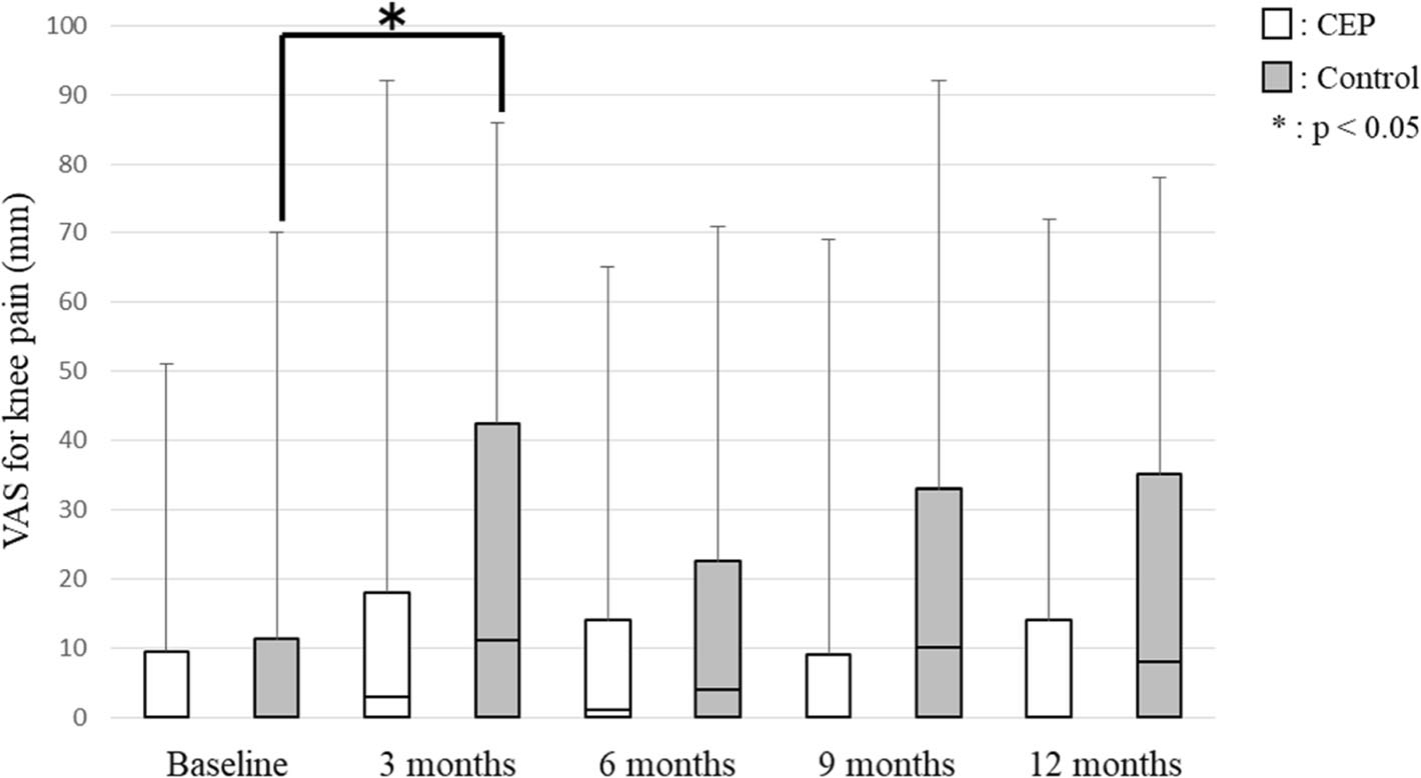
Visual analogue scale (VAS) for knee pain

**Fig. 6:**
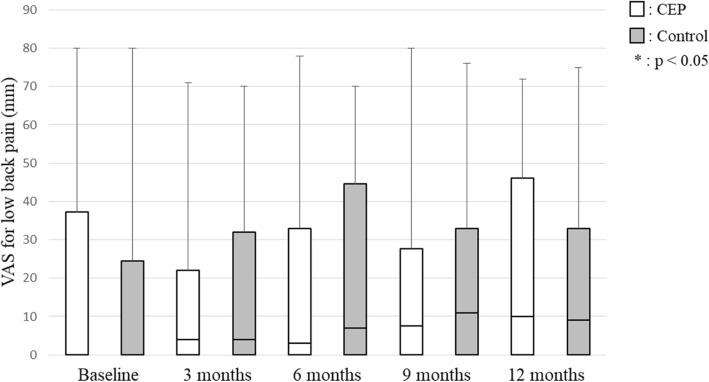
Visual analogue scale (VAS) for low back pain

No significant between-group differences in any secondary outcomes were observed at any time point. SE for exercise improved 24.0% (CEP group 30.8%; control group 16.7%) compared to baseline. SE for exercise showed no significant differences between baseline and each evaluation time point in the CEP group, but it was significantly lower at 9 months (95% confidence interval (CI), − 4.50 – 0.00; *p* = 0.038) and 12 months (95% CI, − 4.00 – − 1.00; *p* = 0.007) in the control group (Fig. 2). The stage of change for exercise behavior was significantly higher at 3 months (95% CI, 0.50–1.50; *p* = 0.001), 6 months (95% CI, 0.50–2.00; *p* = 0.001), 9 months (95% CI, 0.50–2.00; *p* = 0.001) and 12 months (95% CI, 0.50–1.50; *p* = 0.002) compared with baseline in the CEP group (Fig. 3). In the control group, the stage of change for exercise behavior was significantly higher at 6 months (95% CI, 0.50–1.50; *p* = 0.026) and 12 months (95% CI, 0.00–1.50; *p* = 0.039) compared with baseline. Exercise adherence was significantly lower at 9 months (95% CI, − 29.42 – − 7.47; *p* = 0.001) and 12 months (95% CI, − 20.64 – − 1.13; *p* = 0.011) compared with at 3 months, and at 9 months (95% CI, − 19.15 – 2.30; *p* = 0.002) compared with 6 months in the CEP group (Fig. 4). In the control group, exercise adherence was significantly lower at 9 months (95% CI, − 15.83 – − 0.01; *p* = 0.048) than at 3 months. Knee pain was not significantly different between baseline and each evaluation time point in the CEP group, but knee pain was worse at 3 months (95% CI, 0.00–14.50; *p* = 0.009) compared with baseline in the control group (Fig. 5). Low back pain did not show any significant differences between baseline and each evaluation time point in either group (Fig. 6).

## Discussion

In the present study, none of the variables examined were significantly different between the CEP and control groups throughout the 12 months. However, within-group comparisons indicated the appearance of behavioral changes at 3 months (95% CI, 0.50–1.50) in the CEP group and at 6 months (95% CI, 0.50–1.50) in the control group. In the CEP group, SE for exercise did not decrease and there was no exacerbation of knee and low back pain. In the control group, SE for exercise was significantly lower at 9 months (95% CI, − 4.50 – 0.00) and 12 months (95% CI, − 4.00 – − 1.00) and knee pain was worse at 3 months (95% CI, 0.00–14.50) compared with baseline. These results suggested that individualized exercise regimens can be implemented without exacerbation of knee and low back pain and that exercise programs designed with consideration of physical strength and pain levels can encourage community-dwelling elderly individuals who do not exercise regularly to start and continue doing so.

Stages of change for exercise behavior were significantly better at 3 months in the CEP group. In previous studies, the central role of SE in behavioral change [12, 16] and the relationship between the environment and behavioral change [27] were pointed out. Recently, a relationship between behavioral change and perceived health benefits of training or barriers to training has been reported [28]; the distorted perception that strength training cannot be done without going to a facility or that strength training is a strenuous exercise was discussed as a barrier to strength training. The exercise programs prescribed to the CEP group were designed based on the motor function of each subject and could be completed at home, which possibly led to fewer barriers to exercise and caused behavioral change in the subjects.

SE for exercise did not decrease in the CEP group over time. In contrast, SE for exercise was significantly lower at 9 and 12 months compared with baseline in the control group. SE for exercise was reported to be associated with motivation for exercising [29]. To improve SE for exercise, individuals need to recognize the risks they have, expect that exercise will be beneficial, and have successful exercise experiences [29]. By being made aware of their musculoskeletal age*,* which was estimated based on various measurements and values indicating physical strength, the subjects in the CEP group may have recognized the risks of motor dysfunction and expected that these risks would be reduced by exercise. Moreover, in the CEP group, the ease of implementing the exercise programs designed based on individual motor function may have led to successful experiences, which stimulated interest in exercising and contributed to motivation for starting to exercise. In the control group, we speculate that the standardized programs did not motivate the subjects sufficiently, possibly causing a decrease in SE.

Knee and low back pain were not exacerbated in the CEP group. The algorithm used for the CEP group prescribed exercises that do not place a burden on the sites of pain. This is probably the reason why the exercise programs did not cause worsening of knee and low back pain. In contrast, knee pain was significantly worse at 3 months compared with baseline in the control group. This worsening may be related to the fact that squatting was prescribed regardless of whether subjects in the control group had knee pain. Although commonly recommended, squatting may increase the burden on knee joints [30]. Thus, squatting probably increased the burden on knees and worsened knee pain in the control group.

However, by 6 months, exercise adherence in the CEP group had gradually declined compared with baseline and 3 months. Previous studies have shown that approximately 50% of older adults who start new exercise regimens suspend them within 6 months [31] and that the rate of continuing to exercise after attending exercise classes is approximately 30% in older adults [11], indicating the difficulty in continuing to exercise. Another study pointed out the importance of monitoring and providing feedback for exercise adherence [32]. In the present study, monitoring was performed every 3 months, but no feedback was given to the subjects. This lack of feedback may be a cause of the decline in exercise adherence. The results of this study suggest that it is essential to implement regular feedback and review exercise programs in order to improve exercise adherence.

This study has several limitations. One is insufficient blinding. Not blinding the physical therapists who gave instructions to the subjects and the subjects themselves possibly influenced the results. However, a statistician who was not affiliated with the study prepared the allocation table and motor function testing and statistical analysis were performed in a blinded manner to prevent the influence of bias in measurement and analysis. The second limitation is the number of subjects. In planning, recruitment of 60 subjects was anticipated, but only 50 subjects actually participated. Nevertheless, the sample size was not too much smaller than the target and we speculate that the number of subjects did not have a major impact on the results. The third limitation is dropout. The lack of follow-up for some subjects possibly gave rise to withdrawal bias. However, there was only 1 dropout each in the 2 groups, probably because evaluation was performed every 3 months in this study. The fourth limitation is external validity. We performed this study in a small rural town, which may limit the generalizability of the results to urban areas.

## Conclusion

In elderly people who do not exercise regularly, exercise instruction by physical therapists using CEPs contributed to the maintenance of SE for exercise. CEPs were designed based on the subject’s motor function and could be completed at home, which contributed to behavioral changes at 3, 6, 9, and 12 months compared with baseline. This study suggested that individualized exercise regimens can be implemented without exacerbation of knee and low back pain and that exercise programs designed with consideration of physical strength and pain levels can encourage community-dwelling elderly individuals who do not exercise regularly to start and continue doing so.

## Additional file

Additional file 1: CEP group exercise programs (DOCX 16 kb)

## Data Availability

The datasets generated and analyzed during the current study are not publicly available due to professional discretion, as they were part of patient’s records, but are available as a de-identified data sheet from the corresponding author on reasonable request.

## Abbreviations

CEP: Customized exercise program
GAINA study: Good Ageing and Intervention Against Nursing Care and Activity Decline study
SE: Self-efficacy
VAS: Visual analogue scale
YAM: Young Adult Mean
