# Supplementary material for: Customized exercise programs implemented by physical therapists improve exercise-related self-efficacy and promote behavioral changes in elderly individuals without regular exercise: a randomized controlled trial
Source: BMC Public Health. 2019 Jul 9;19:917. doi: 10.1186/s12889-019-7270-7 (PMC6617696; doi:10.1186/s12889-019-7270-7)
Supplement: Supplementary file 1 — CEP group exercise programs (DOCX 16 kb) [file 12889_2019_7270_MOESM1_ESM.docx]

**Additional file 1** CEP group exercise programs

| No. | Exercise 1 | Exercise 2 | Exercise 3 |
| --- | --- | --- | --- |
| 1 | Hamstrings stretch (30sec, 2sets) | Heel raise (30sec, 2sets) | Squat (15times, 2sets) |
| 2 | Straight leg raising (20times, 1set) | Pelvis exercise (30times, 1set) |  |
| 3 | Trunk extension exercise (5times, 1set) | Squat (20times, 2sets) |  |
| 4 | One leg standing (30sec, 2sets) | Standing exercise (15times, 2sets) | Walking (15min) |
| 5 | Lumbar stretch (30sec, 2times) | Foot stepping exercise (1min, 2sets) | Achilles tendon stretch (30sec, 2sets) |
| 6 | One leg standing (30sec, 2sets) | Squat (20times, 2sets) |  |
| 7 | Trunk extension exercise (5times, 1set) | Walking (15min) |  |
| 8 | Squat (15times, 2sets) | Walking (15min) |  |
| 9 | Straight leg raising (15times, 1set) | Heel raise (30sec, 2sets) | Pelvis exercise (30times, 2set) |
| 10 | Pelvis exercise (20times, 3set) | Walking (15min) |  |
| 11 | Trunk extension exercise (5times, 1set) | Foot stepping exercise (1min, 2sets) | Standing exercise (15times, 2sets) |
| 12 | Straight leg raising (15times, 2sets) | Foot stepping exercise on sitting (3min, 3sets) |  |
| 13 | Straight leg raising (15times, 2sets) | Foot stepping exercise on sitting (20times, 2sets) | Pelvis exercise (30times, 2set) |
| 14 | Quadriceps stretch (30sec, 2sets) | Walking (20min) |  |
| 15 | Trunk extension exercise (5times, 1set) | Foot stepping exercise (1min, 2sets) | Squat (15times, 2sets) |
| 16 | Straight leg raising (15times, 2sets) | Heel raise (30sec, 2sets) | Walking (15min) |
| 17 | Side foot stepping exercise (20times, 2sets) | Quadriceps stretch (30sec, 2sets) |  |
| 18 | Walking (15min) |  |  |
| 19 | Straight leg raising (20times, 1set) | Trunk extension exercise (5times, 1set) | Heel raise (30sec, 2sets) |
| 20 | Straight leg raising (20times, 1set) | Pelvis exercise (30times, 1set) | Heel raise (30sec, 2sets) |
| 21 | Trunk extension exercise (5times, 1set) | Walking (15min) | Side foot stepping exercise (20times, 2sets) |
| 22 | Squat (15times, 2sets) | One leg standing (30sec, 2sets) |  |
| 23 | Foot stepping exercise (1min, 2sets) | Standing exercise (20times, 2sets) | Squat (20times, 2sets) |
| 24 | Foot stepping exercise (1min, 2sets) | Walking (20min) |  |
| 25 | Walking (20min) | Standing exercise (20times, 2sets) |  |
| 26 | Trunk extension exercise (5times, 1set) | Standing exercise (20times, 2sets) |  |
